# Effect of Ulinastatin on Syndecan-2-Mediated Vascular Damage in IDH2-Deficient Endothelial Cells

**DOI:** 10.3390/biomedicines10010187

**Published:** 2022-01-17

**Authors:** Su-jeong Choi, Harsha Nagar, Jun Wan Lee, Seonhee Kim, Ikjun Lee, Shuyu Piao, Byeong Hwa Jeon, Cuk-Seong Kim

**Affiliations:** 1 Department of Physiology & Medical Science, College of Medicine, Chungnam National University, Daejeon 301-747, Korea; 01030028473@naver.com (S.-j.C.); harsha_nagar2002@yahoo.com (H.N.); wlxlsunny@naver.com (S.K.); tw2622@gmail.com (I.L.); piaoshuyu@cnu.ac.kr (S.P.); bhjeon@cnu.ac.kr (B.H.J.); 2 Emergency ICU, Regional Emergency Center, Chungnam National University Hospital, Daejeon 301-747, Korea; u2lee@hanmail.net

**Keywords:** ulinastatin, IDH2, SDC2, endothelial cells, vascular damage

## Abstract

Syndecan-2 (SDC2), a cell-surface heparin sulfate proteoglycan of the glycocalyx, is mainly expressed in endothelial cells. Although oxidative stress and inflammatory mediators have been shown to mediate dysfunction of the glycocalyx, little is known about their role in vascular endothelial cells. In this study, we aimed to identify the mechanism that regulates SDC2 expression in isocitrate dehydrogenase 2 (IDH2)-deficient endothelial cells, and to investigate the effect of ulinastatin (UTI) on this mechanism. We showed that knockdown of IDH2 induced SDC2 expression in human umbilical vein endothelial cells (HUVECs). Matrix metalloproteinase 7 (MMP7) influences SDC2 expression. When IDH2 was downregulated, MMP7 expression was increased, as was TGF-β signaling, which regulates MMP7. Inhibition of MMP7 activity using MMP inhibitor II significantly reduced SDC2, suggesting that IDH2 mediated SDC2 expression via MMP7. Moreover, expression of SDC2 and MMP7, as well as TGF-β signaling, increased in response to IDH2 deficiency, and treatment with UTI reversed this increase. Similarly, the increase in SDC2, MMP7, and TGF-β signaling in the aorta of IDH2 knockout mice was reversed by UTI treatment. These findings suggest that IDH2 deficiency induces SDC2 expression via TGF-β and MMP7 signaling in endothelial cells.

## 1. Introduction

The glycocalyx is a carbohydrate-based coating that covers the outer surface of the cell membrane, including the transmembrane glycoproteins that protect the cell. This coating acts as a barrier between the cell and its surroundings and is also a mediator of cell–cell interactions. The glycocalyx protects cell membranes from the direct action of physical forces and stress and is also involved in the initiation and progression of many diseases, including atherosclerosis, kidney disease, hypertension, pulmonary edema, stroke, sepsis, and other diseases [1]. Discoveries pertaining to the glycocalyx can lead to new treatment modalities for various diseases, in particular vascular diseases [2]. The glycocalyx protects the endothelial cell surface and inhibits inflammation by preventing the adhesion of leukocytes or inflammatory cytokines. It also supports vascular transport barriers and mediates important transformation processes such as flow-mediated vasodilation [3,4]. Glycocalyx dysfunction has been shown to occur in response to oxidative stress and inflammatory mediators [5,6]. The glycocalyx is composed of glycosaminoglycans, proteoglycans, and glycoproteins. Most glycocalyx-related proteins penetrate cell membranes and attach to the cytoskeleton. This linkage is structured not only to limit the position and form the basis of the glycocalyx structure but also to facilitate signal transduction across the cell membrane [7,8]. Syndecan (SDC) is a glycoprotein of the glycocalyx that is tightly bound through the membrane-spanning domain [9]. SDC is associated with angiogenesis, inflammation, and cancer [10,11,12]. There are four SDCs: SDC1, SDC2, SDC3, and SDC4. Among them, SDC2 is highly expressed in endothelial cells, but exhibits low levels of expression in fibroblasts, neurons, and smooth muscle cells [13].

The TGF-β signaling pathway is involved in many cellular processes, including cell growth, cellular homeostasis, cell differentiation, apoptosis, and other cellular functions in both the developing embryo and adults. TGF-β mediates the induction of many matrix structural proteins, such as MMPs and pro-fibrotic mediators [14]. Matrix metalloproteinases (MMPs) comprise a large group of extracellular proteins that belong to the metalloproteinase super family [15]. In general, MMPs are expressed in active cells and play important roles in processes such as degradation of the extracellular matrix, proteolytic digestion of secreted proteins, and movement of cells [16,17]. Glycocalyx dysfunction has been attributed to tissue remodeling MMPs, including MMP1, MMP2, MMP7, MMP9, MMP12, and MMP14, which have been largely investigated in vascular research. The functions of MMPs and cell surface receptors are closely related. For example, MMP7 was shown to interact with SDC2 in colon cancer cells [18,19]. However, the correlation between SDC2 and MMP7 in endothelial cells has not yet been elucidated.

Ulinastatin (UTI) is a glycoprotein that belongs to a broad range of serine protease inhibitors isolated from human urine. UTI downregulates the expression of inflammatory cytokines and is used for the treatment of acute circulatory disorders [20]. UTI also displays powerful anti-inflammatory and cell protective effects in various cell and animal models [21]. Furthermore, UTI attenuates reactive oxygen species (ROS) transfer between neighboring human umbilical cord vascular endothelial cells (HUVECs), resulting in downregulation of the JAK2/STAT3 signaling pathway and its downstream MMP2 and MMP9 effectors [22].

Isocitrate dehydrogenase 2 (IDH2) is an essential mitochondrial NADP+-dependent enzyme that defends the cell against oxidative stress. It catalyzes the conversion of isocitrate into α-ketoglutarate in the TCA cycle, resulting in the generation of NADPH, which plays an important role in the antioxidant system and in the regeneration of the reduced glutathione pool [23]. We previously showed that IDH2 downregulation resulted in mitochondrial dysfunction, ROS generation, and inflammation induction in endothelial cells [24]. However, the mechanism by which mitochondrial dysfunction induced by IDH2 deficiency mediates SDC2 expression in vascular endothelial cells is unknown. Moreover, there are no previous studies to explain the relationship of Isocitrate dehydrogenases with syndecans. Therefore, in this study, we tried to identify the mechanism that regulates the expression of SDC2 and MMP7 in IDH2-deficient endothelial cells, and the role of UTI in this process.

## 2. Experimental Section

### 2.1. Cell Culture and Transfection

HUVECs were obtained from the American Type Culture Collection (Manassas, VA, USA) and cultured in Endothelial Growth Medium-2 (Lonza, Walkersville, MD, USA). Sub-confluent, proliferating HUVECs were used at passages four to eight. HUVECs were transfected with human IDH2 short interfering RNA (siRNA): sense-CAGUAUGCCAUCCAGAAGA (dTdT) and antisense-UCUUCUGGAUGGCAUACUG (dTdT) (Bioneer, Daejeon, South Korea) and negative control siRNA using Lipofectamine 2000 reagent (Invitrogen, Carlsbad, CA, USA) following the manufacturer’s recommendations.

### 2.2. Mouse Studies

All animal experiments were approved and carried out in accordance with the guidelines of the Animal Care Committee of Chungnam National University (CNU-00084). The animals used in this study were IDH2−/− germ-line knockout (IDH2 KO) mice; their congenic background strain (C57BL/6) served as the wild-type (WT, IDH2+/+) control. Animals were raised in controlled lighting (from 6:00 a.m. to 6:00 p.m., daily) and temperature (24 ± 1 °C) conditions with free access to food and water. IDH2 KO mice were a kind donation from Kyungpook National University (School of Life Sciences, College of Natural Science, South Korea). All instruments were used under aseptic conditions. Mice were divided into four groups: WT, WT+UTI, IDH2 KO and IDH2 KO+UTI (n = 10 mice/group). C57BL/6 mice were injected intraperitoneally with UTI (2 × 10^4^ U/kg) (WT+UTI, IDH2 KO+UTI). For sham control, mice were injected intraperitoneally with saline (WT, IDH2 KO). UTI solution and saline were administered twice in 24 h (every 12 h). Twenty-four house after administration of UTI, mice were sacrificed by an overdose of urethane anesthesia. A mid-sternal split was quickly performed, and the descending thoracic aorta was carefully excised and snap frozen for subsequent western blot analysis.

### 2.3. Immunocytochemistry

HUVECs were cultured on glass coverslips and transfected with si-IDH2 for 48 h. After washing with phosphate-buffered saline (PBS), cells were fixed with 4% paraformaldehyde and permeabilized with 0.25% Triton X-100. After blocking for 30 min with PBS containing 1% bovine serum albumin, cells were incubated with SDC2 antibody (1:500) overnight at 4 °C and labeled with fluorescein isothiocyanate-conjugated secondary antibody (1:500) for 1 h in the dark at room temperature. Images were obtained by fluorescence microscopy.

### 2.4. Antibodies and Immunoblotting

The following antibodies were used: IDH2 (Abcam, Cambridge, UK), SDC2 (Invitrogen), MMP7 (Santa Cruz Biotechnology, Santa Cruz, CA, USA), β-catenin, TGF-β, phospho-Smad2, Total-Smad2, phospho-Smad3, Total-Smad3 (Cell Signaling, Beverly, MA, USA), and β-actin (Sigma Aldrich, Darmstadt, Germany). Whole cell lysates or tissue homogenates from 8-weeks-old sham control or WT and IDH2 KO mice treated with UTI (20 μg) were loaded and separated on 9%–15% sodium dodecyl sulfate polyacrylamide gel electrophoresis gels, followed by incubation with the appropriate primary and secondary antibodies. Blots were imaged using a chemiluminescence assay kit (Thermo Scientific, Rockford, IL, USA) and band densities were quantified on a Gel Doc 2000 Chemi Doc system using Quantity One software (Bio-Rad, Hercules, CA, USA). Values were normalized to β-actin (loading control).

### 2.5. Real-Time Polymerase Chain Reaction (qPCR)

Total RNA was isolated from the cells using TRIzol reagent (Thermo Fisher Scientific, Waltham, MA, USA). Complementary DNA (cDNA) was generated using an RT premix kit (CellScript All-in-One 5X First Strand cDNA Synthesis Master Mix; CellSafe, Gyeonggi City, South Korea). Relative RNA expression levels were determined by PCR using SYBR qPCR premix (Enzynomics, Daejeon, South Korea). Primer sequences used for quantitative PCR are summarized in Supplementary Table S1. The PCR cycling conditions were as follows: 5 min at 95 °C, 40 cycles of 30 s at 95 °C, 30 s at 60 °C, and 30 s at 72 °C, followed by 5 min at 72 °C. GAPDH was used as an internal control. Results were interpreted using the relative quantity method (ΔΔCt).

### 2.6. Cell Viability Assay

Cell viability after dose and time-dependent UTI treatment in HUVECs, was measured using an ADAM-MC automatic cell counter AccuChip kit (Digital Bio., Seoul, South Korea) which is based on a fluorescent microscopy technique for counting cells. It functions by using the propidium iodide (PI) staining method of dead cell staining. Cells were treated with UTI (30, 100, 300 and 1000 U/mL) for 24, 48, 72 and 96 h and cell counting measurement was performed according to the manufacturer’s instructions.

### 2.7. CCK-8 Proliferation Assay

Cell proliferation was measured using the Cell Counting Kit-8 (CCK-8) (Dojindo, Rockville, MD, USA) according to the manufacturer’s instructions. Briefly, cells transfected with negative control or IDH2 siRNA with and without UTI treatment for 48 h were washed with PBS and resuspended in growth medium including 25 µL CCK-8 solution and incubated for 1 h at 37 °C in a 5% CO_2_ humidified incubator, followed by measurement of absorbance at 450 nm.

### 2.8. Cell Scratch Assay

A scratch assay was used to assess cell migration. Cells were transfected as described previously with negative control or IDH2 siRNA with and without UTI treatment in six-well tissue culture plates for 24 h, after which a sterile 200 μL pipette tip was used to detach the cells from the monolayer across the center of the well. Floating cells were flushed out by gently rinsing twice with PBS and replaced with serum-free medium (to rule out cell proliferation as the cause of wound closure) followed by incubation for another 24 h. The total incubation time post transfection was therefore 48 h. Cell movement was monitored using microscopy. Photographs were taken immediately and at 24 h after scratching. The relative wound area was quantitatively evaluated using ImageJ software (NIH, Bethesda, MD, USA).

### 2.9. Mouse Genotyping

Chromosomal DNA was isolated from mouse tails (2–3 mm length). PCR was conducted using a KOD-FX genotyping kit (TOYOBO, Iwakuni, Japan) according to the manufacturer’s instructions. The primer sequences were as follows: reverse (5′-CCAGTCATAGCCGAATAGCC-3′), forward (5′-GATAGAATTTCCGTGGCAAC-3′) for IDH2 KO; and forward (5′-ACTGTTCTGGAACATGCTGC-3′), reverse (5′TCCTCAAAGCATCAGGTACC-3′) for IDH2 WT. PCR cycle conditions were as follows: 95 °C for 5 min (one cycle), 95 °C for 30 s, 60 °C for 45 s, 72 °C for 1 min (30 cycles), and 72 °C for 10 min.

### 2.10. Statistical Analysis

Statistical analysis was performed using GraphPad Prism 6 (GraphPad Software Inc., San Diego, CA, USA). Data were expressed as the means ± standard error of mean. Differences between two groups were evaluated using *t*-tests. For multiple comparisons, one-way analysis of variance was performed followed by an appropriate multiple comparison test. *p*-values less than 0.05 were considered statistically significant. All data were representative of at least three independent experiments.

## 3. Results

### 3.1. Expression of SDC2 Is Increased in IDH2-Deficient HUVECs

**Figure 1:**
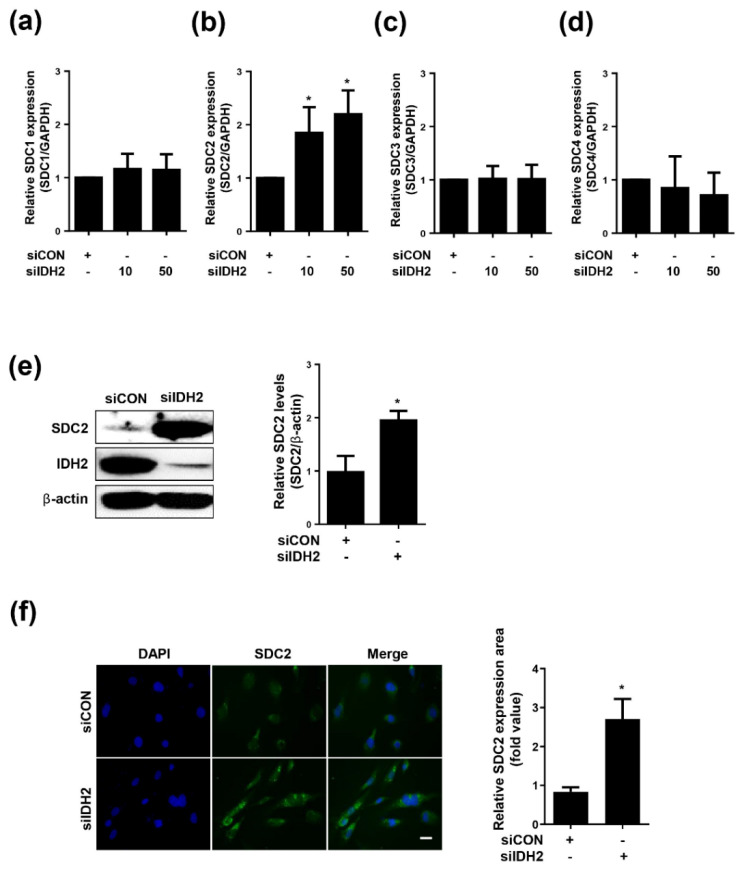
Expression of Syndecans in IDH2 deficient HUVECs. HUVECs were transfected with dose dependent siIDH2 (10 pmol, 50 pmol) and siCON for 48h. mRNA expressions of (**a**) SDC1, (**b**) SDC2, (**c**) SDC3, (**d**) SDC4 were quantified by qPCR (**e**) protein level of SDC2 was detected by western blotting. β-actin was used as a loading control. Protein levels were quantified by densitometric analysis using Image-J software (Shown in the right panel). (**f**) Immunofluorescence analysis of HUVECs to compare the SDC2 expression in siCON and siIDH2 (50 pmol) transfected HUVECs (scale bar 100 μm). All data are presented as means ± SEM of three independent experiments, * *p* < 0.05, compared with siCON.

To investigate the relationship between IDH2 and SDC, we measured the mRNA expression of SDC1, 2, 3, and 4 following downregulation of IDH2. Only SDC2 showed significantly increased mRNA levels (Figure 1a–d). Furthermore, only SDC2 protein level was increased upon knockdown of IDH2 (Figure 1e). Fluorescence expression of SDC2 in HUVECs was confirmed by immunocytochemistry staining. SDC2 fluorescence was increased in the IDH2 knockdown condition compared to the control group (Figure 1f). Overall, these results suggest that IDH2 deficiency affects SDC2 expression.

### 3.2. TGF-β and MMP7 Signaling Correlate with SDC2 Expression in IDH2-Deficient HUVECs

**Figure 2:**
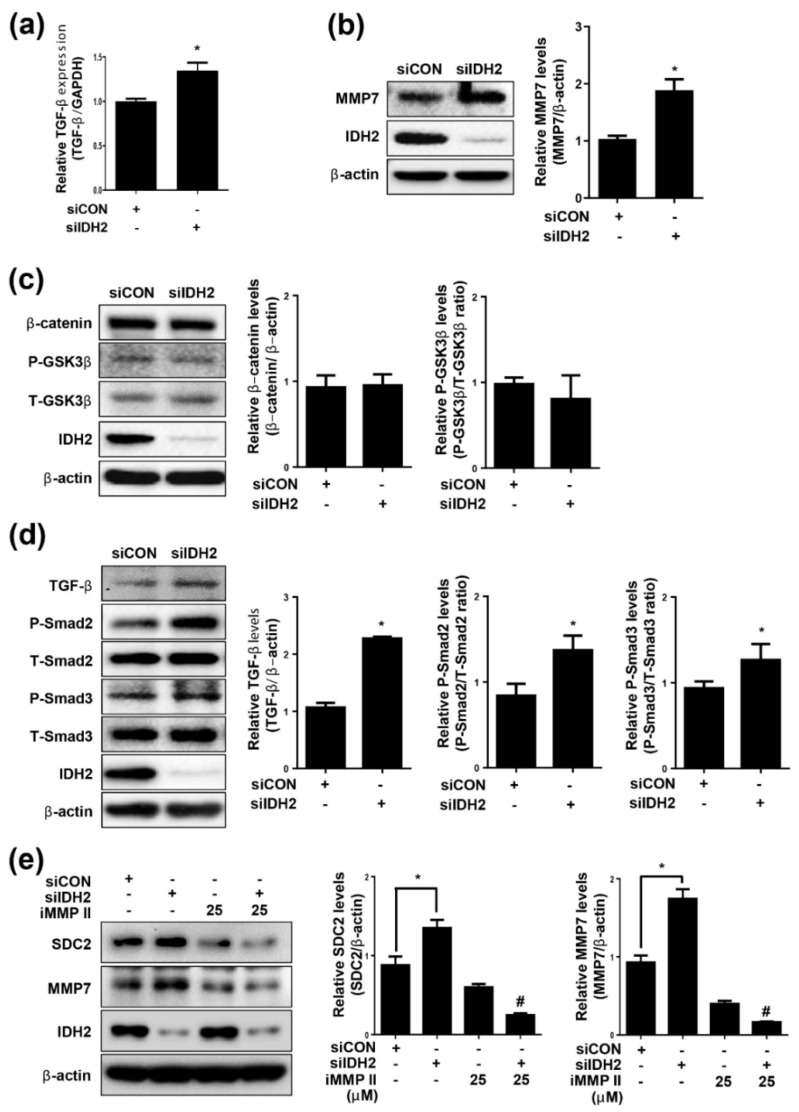
Involvement of TGF-β and MMP7 signaling in relation to SDC2 shedding in IDH2 deficient HUVECs. HUVECs were transfected with 50 pmol of siIDH2 and siCON for 48 h. (**a**) mRNA expression of TGF-β was quantified by qPCR. Protein levels of (**b**) MMP7 (**c**) β-catenin and (**d**) TGF-β signaling-related proteins were detected by western blotting. β-actin was used as a loading control. Protein levels were quantified by densitometric analysis using Image-J software (Shown in the right panel). HUVECs were transfected with 50 pmol of siIDH2 and siCON, incubated for 24 h and treated with MMP inhibitor II (25 μM) for another 24 h. Protein levels of (**e**) MMP7 and SDC2 were detected by western blotting. β-actin was used as a loading control. Protein levels were quantified by densitometric analysis using Image-J software (Shown in the right panel). All data are presented as means ± SEM of three independent experiments, * *p* < 0.05, compared with siCON, ^#^ *p* < 0.05 compared with the 50 pmol siIDH2 treated cells.

MMPs are associated with extracellular matrix protein degradation and cell surface receptor cleavage. To determine which MMP affected SDC2 expression under IDH2 deficiency, we screened various MMP subtypes. Only MMP7 mRNA expression was significantly increased in IDH2 deficient cells (Supplementary Figure S1). The protein level of MMP7 was also significantly increased in IDH2-deficient HUVECs (Figure 2b). These findings suggest that MMP7 was involved in the expression of SDC2 under IDH2 knockdown conditions.

Signaling pathways that regulate MMP7 include β-catenin and TGF-β signaling. The Wnt/β-catenin pathway is a conserved signaling pathway that has a fundamental role in regulating various biological processes, such as pathogenesis of human diseases, tissue homeostasis, and organ development. Moreover, it is well established that TGF-β regulates MMP7 expression [25]. In the present study, although there was no change in the β-catenin pathway (Figure 2c), TGF-β mRNA expression (Figure 2a) and protein level (Figure 2d) as well as downstream signaling were increased under IDH2-deficient conditions.

Several MMPs are involved in SDC cleavage in vitro and in vivo [26]. In this study, we demonstrated that silencing of MMP7 with an MMP inhibitor (MMP inhibitor II) significantly attenuated SDC2 level (Figure 2e). This finding suggests that downregulation of MMP7 led to a decrease in IDH2 deficiency-induced SDC2 expression in HUVECs.

### 3.3. Effect of UTI on SDC2, MMP7, and TGF-β Signaling in IDH2-Deficient HUVECs

**Figure 3:**
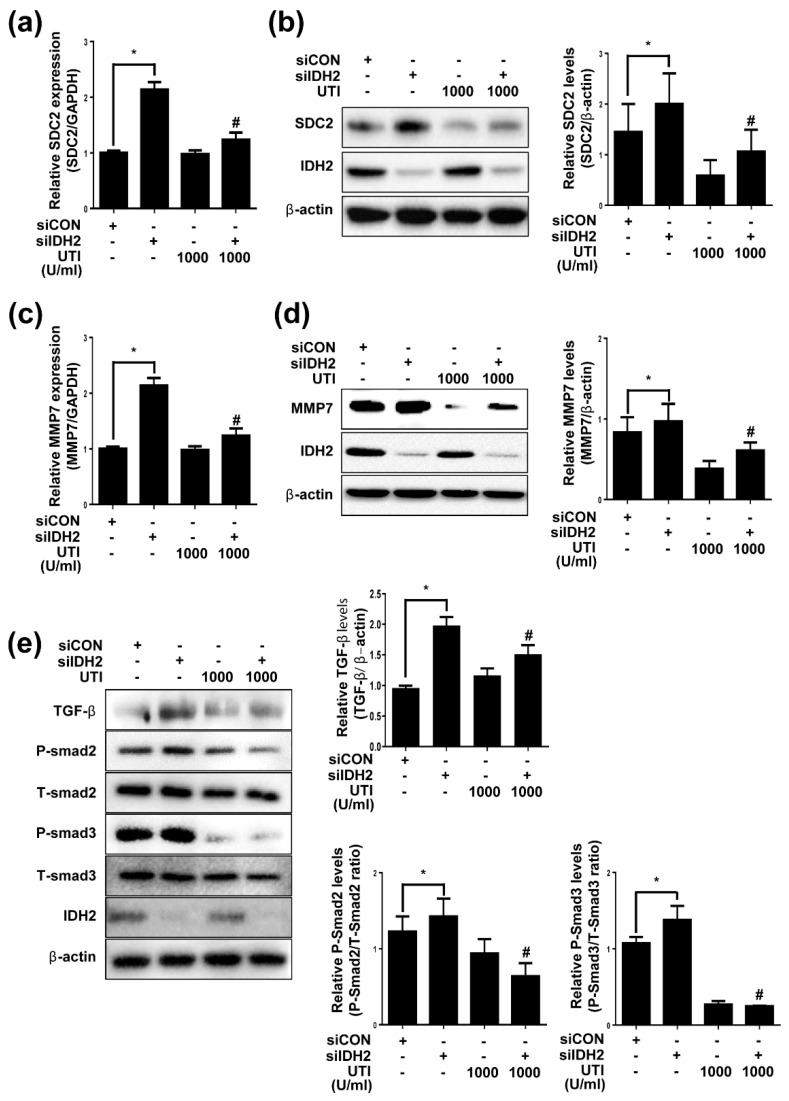
Effect of UTI on SDC2, MMP7 and TGF-β signaling in IDH2 deficient HUVECs. HUVECs were transfected with 50 pmol of siIDH2 and siCON and 1000 U/mL of UTI. mRNA expressions of (**a**) SDC2 and (**c**) MMP7 were quantified by qPCR. Protein levels of (**b**) SDC2 and (**d**) MMP7 were detected by western blotting. (**e**) Protein level of TGF-β signaling was detected by western blotting. β-actin was used as a loading control. Protein levels were quantified by densitometric analysis using Image-J software (Shown in the right panel). All data are presented as means ± SEM of three independent experiments, * *p* < 0.05, compared with siCON, ^#^ *p* < 0.05 compared with the 50 pmol siIDH2 treated cells.

UTI is a serine protease inhibitor with many biological activities, including anti-inflammatory and antioxidant effects. We used UTI to treat HUVECs in dose- and time-dependent manners, and measured cell viability (Supplementary Figure S2a). As there was no significant difference in response to dose and time, we selected 1000 U/mL and 48 h for all subsequent experiments. We transfected HUVECs with siIDH2 with or without the treatment of UTI, and measured cell proliferation (Supplementary Figure S2b), cell migration (Supplementary Figure S3a), and markers of cell proliferation (PCNA), angiogenesis (MMP9), apoptosis (cleaved caspase 9) (Supplementary Figure S3b). As shown in the results, UTI had no effect on cell proliferation, but it increased cell migration and decreased cell apoptosis. The increase in SDC2 mRNA and protein observed in IDH2-deficient HUVECs was reversed by UTI treatment (Figure 3a,b), suggesting that UTI had a therapeutic effect related to SDC2 expression under IDH2 deficiency. In addition, we investigated the effect of UTI on MMP7 and TGF-β signaling. UTI treatment reversed the increase in MMP7 mRNA and protein (Figure 3c,d), along with TGF-β mRNA (Supplementary Figure S4a) and protein (Figure 3e) as well as downstream signaling in IDH2-deficient HUVECs. Next, we measured the expression of inflammatory cytokines TNF-α and IL-1β (Supplementary Figure S4b–d). The increase in inflammatory cytokines in IDH2-deficient HUVECs was reduced by UTI treatment. These results suggest that UTI has a therapeutic effect on TGF-β signaling and MMP7 expression increased by IDH2 deficiency in HUVECs.

### 3.4. Effect of UTI on SDC2, MMP7, and TGF-β Signaling in Aorta from IDH2 KO Mice

**Figure 4:**
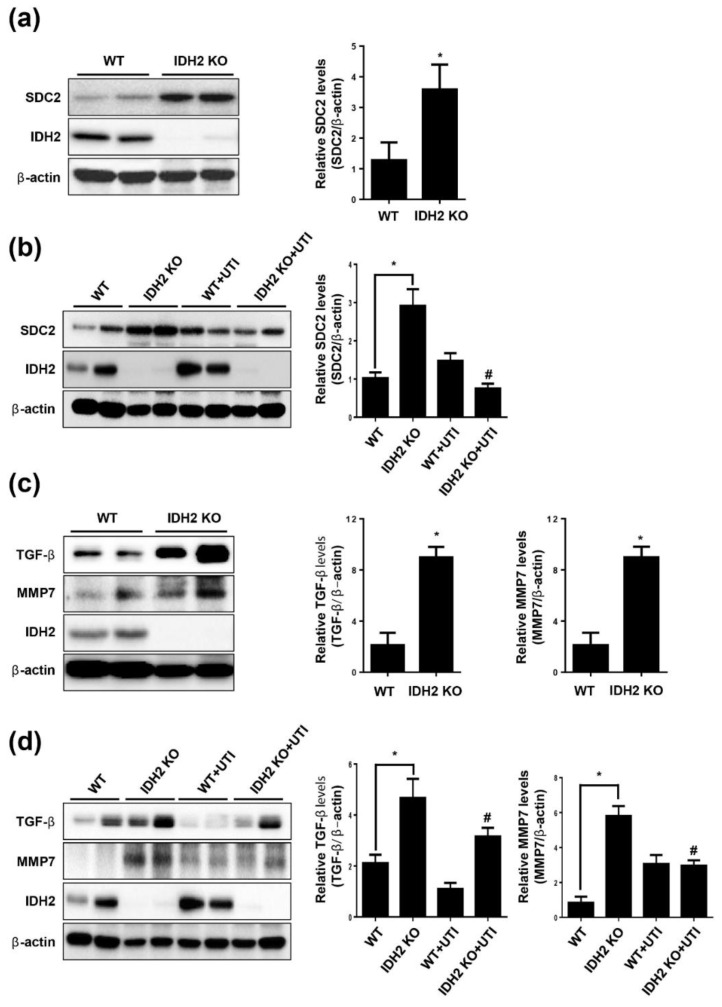
Effect of UTI on SDC2, MMP7 and TGF-β signaling in aorta from IDH2 KO mice. Aorta were isolated from WT and IDH2 KO mice and after peritoneal injection of UTI in WT and IDH2 KO mice. (**a**,**b**) Protein level of SDC2, (**c**,**d**) TGF-β and MMP7 were detected by western blotting. β-actin was used as a loading control. Protein levels were quantified by densitometric analysis using Image-J software (Shown in the right panel). All data are presented as means ± SEM, * *p* < 0.05 compared with WT mice, ^#^ *p* < 0.05 compared with UTI injected IDH2 KO mice (n = 10 mice/group).

To examine the effect of IDH2 KO on SDC2 expression in vivo, we isolated aortae from WT and IDH2 KO mice and then measured SDC2 expression. SDC2 protein level in IDH2 KO mice was significantly increased compared with those in WT mice (Figure 4a). Next, we investigated how UTI affected SDC2 in vivo. Following administration of UTI, aortae were isolated from IDH2 KO and WT mice. UTI treatment decreased SDC2 expression, counteracting the initial increase in expression observed in IDH2 KO mice (Figure 4b). To determine the effect of IDH2 KO on MMP7 and TGF-β in vivo, we measured MMP7 and TGF-β levels in the aortae isolated from WT and IDH2 KO mice. MMP7 and TGF-β in IDH2 KO mice were significantly increased compared with those in WT mice (Figure 4c). Next, to determine how UTI affected MMP7 and TGF-β in vivo, following administration of UTI, aortae were isolated from IDH2 KO and WT mice. The increased level of MMP7 and TGF-β initially observed in IDH2 KO was reversed by UTI treatment (Figure 4d).

## 4. Discussion

SDC2 is a glycocalyx protein that is highly expressed in endothelial cells. Upregulation of SDC2 expression in endothelial cells during inflammation has been well documented. In addition to vascular inflammation, SDC2 has been associated with vascular diseases and angiogenesis [27]. In our previous study, we have shown that mitochondrial dysfunction in IDH2 deficiency condition ultimately leads to endothelial dysfunction as demonstrated by impaired vascular function [28]. Therefore, we aimed to explore the role of SDC2 in IDH2 deficiency condition to know whether it mediates the mitochondrial dysfunction induced changes in endothelial functions. In this study, we demonstrated that IDH2 knockdown led to an increase in SDC2 expression, which in turn affected vascular endothelial function. The findings of this study indicate that cell damage caused by IDH2 deficiency played a role in controlling the glycocalyx, SDC2 expression, or SDC2 activity.

Several proteins, including MMPs, regulate SDC2. MMPs are proteases in the metzincin superfamily. The MMP family is involved in various mechanisms, including cell proliferation, migration, differentiation, angiogenesis, and apoptosis. In particular, MMPs play roles in processing bioactive molecules and degrading various extracellular matrix proteins. Studies have demonstrated a correlation between SDC2 and MMPs [29]. We screened several MMPs involved in the degradation of extracellular matrix proteins in IDH2-deficient HUVECs to identify the MMP that regulates SDC2 in vascular endothelial cells. After checking the mRNA expressions of MMP2, 3, 7, 9, 12, and 14, and ADAM17, we confirmed that only MMP7 mRNA was significantly increased. Two signaling pathways regulate SDC2 and MMP7: β-catenin and TGF-β pathways [30]. β-catenin signaling plays an important role in the formation of embryonic vascular morphology and has been shown to induce significant changes in endothelial cells and vascular development [31]. TGF-β is a multifunctional cytokine involved in the regulation of various cellular processes such as cell growth, cell differentiation, cell death, and cell homeostasis, as well as the maintenance of tissue homeostasis and tissue repair in adults [32]. We investigated the effect of IDH2 KO on both signaling pathways and found that β-catenin signaling did not change significantly but TGF-β signaling was increased. Next, we used MMP inhibitor II, which inhibits MMP7 expression, to explore the correlation between SDC2 and MMP7 in IDH2-deficient vascular endothelial cells. We confirmed that SDC2 expression, which was increased by IDH2 deficiency, was reduced by MMP7 inhibitor treatment.

UTI is a serine protease inhibitor that inhibits trypsin and is used clinically for the treatment of acute inflammatory reactions such as pancreatitis, disseminated intravascular coagulation, shock, and sepsis [33]. UTI attenuates vascular endothelial cell damage and vascular barrier dysfunction in pregnant women with severe preeclampsia [34,35]. This study demonstrated the recovery effect of cells damaged by IDH2 deficiency through UTI treatment. We demonstrated that UTI moderates the damage caused by IDH2 deficiency via the TGF-β/MMP7 signaling pathway. It has been shown in previous studies, that UTI can markedly reduce the expression and production of TGF-β [36,37]. Consistent with these data, we confirmed that mRNA and protein levels of TGF-β increased under IDH2 deficiency were reduced by UTI treatment. The mRNA and protein levels of the downstream targets of TGF-β signaling (MMP7 and SDC2) which were increased in IDH2 deficiency were similarly reduced by UTI treatment. Thus, we speculate that UTI plays a role in reversing the IDH2 deficiency-induced changes in endothelial cells, although further studies are necessary to test this. Similar results were obtained in the in vivo studies. SDC2 level was increased in the aortae of IDH2 KO mice. After intraperitoneal administration of UTI into IDH2 KO mice, SDC2 protein level, which was increased in the IDH2 KO mice, was reduced by UTI treatment. TGF-β signaling and MMP7 were also increased in the aortae of IDH2 KO mice, and treatment with UTI reversed this increase.

## 5. Conclusions

**Figure 5:**
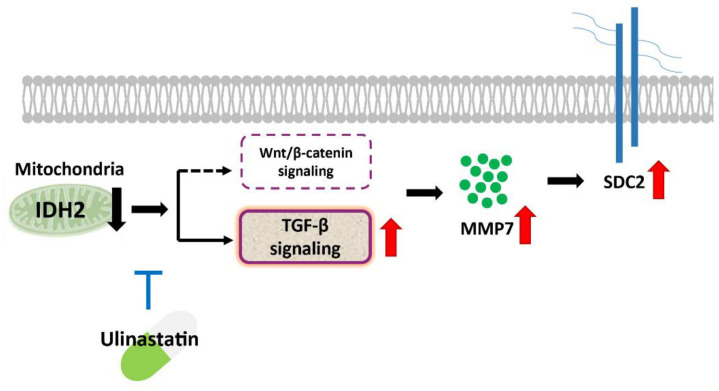
Schematic representation of the proposed pathway of SDC2 activation in IDH2 deficiency condition.

In conclusion, the current study uncovered evidence that the expression level of the core protein SDC2, one of the most abundant heparin sulfate proteoglycans in endothelial cells, is regulated by IDH2, and that this regulation is altered by UTI treatment (Figure 5). This study also demonstrated the molecular biological mechanisms by which UTI reverses the changes induced by IDH2 deficiency in vascular endothelial cells, likely via the TGF-β/MMP7 signaling pathway. However, future investigations are necessary to shed a light on more detailed mechanism and pathway involved in the functioning of UTI in IDH2 deficiency condition.

## Data Availability

The data presented in this study are available on request from the corresponding author.

## Supplementary Materials

The following are available online at https://www.mdpi.com/article/10.3390/biomedicines10010187/s1, Table S1: Primer sequences used in qPCR analyses, Figure S1: Screening of MMP subtypes in HUVECs, Figure S2: Cell viability and cell proliferation after UTI treatment in HUVECs, Figure S3: Measurement of cell function after UTI treatment in HUVECs, Figure S4: Measurement of cytokines after UTI treatment in HUVECs.
