# Supplementary material for: Effect of Ulinastatin on Syndecan-2-Mediated Vascular Damage in IDH2-Deficient Endothelial Cells
Source: Biomedicines. 2022 Jan 17;10(1):187. doi: 10.3390/biomedicines10010187 (PMC8774120; doi:10.3390/biomedicines10010187)
Supplement: Supplementary file 1 [file biomedicines-10-00187-s001.zip › biomedicines-1554039-supplementary.pdf]

Supplementary Table S1

Gene	Forward Sequence (5’-3’)	Reverse Sequence (5’-3’)	Species
IDH2	CCGTGGTGTTCAGGAAGT	GAAGGTGTGCGTGGAGAC	Human
GAPDH	AGGTCGGTGTGAACGGATTTG	GGGGTCGTTGATGGCAACA	Human
MMP7	GAGTGAGCTACAGTGGGAACA	CTATGACGCGGGAGTTTAACAT	Human
SDC1	CTGCCGCAAATTGTGGCTAC	TGAGCCGGAGAAGTTGTCAGA	Human
SDC2	TTGACAACAGCTCCATTGAAGAA	CAGCTCTGGACTCTCTACATCC	Human
SDC3	TGGCGCAGTGAGAACTTCG	CCCCGAGTAGAGGTCATCCAG	Human
SDC4	TCCCCACCGAACCCAAGAA	CCTTGTTGGACACATCCTCAC	Human
TGF-β	CAATTCCTGGCGATACCTCAG	GCACAACTCCGGTGACATCAA	Human
TNF-α	CCCAGGGACCTCTCTCTAATCA	AGCTGCCCCTCAGCTTGAG	Human
IL-1β	CCCAGGGACCTCTCTCTAATCA	AGCTGCCCCTCAGCTTGAG	Human

Supplementary Table 1. Primer sequences used in qPCR analyses.

Supplementary Figure S1

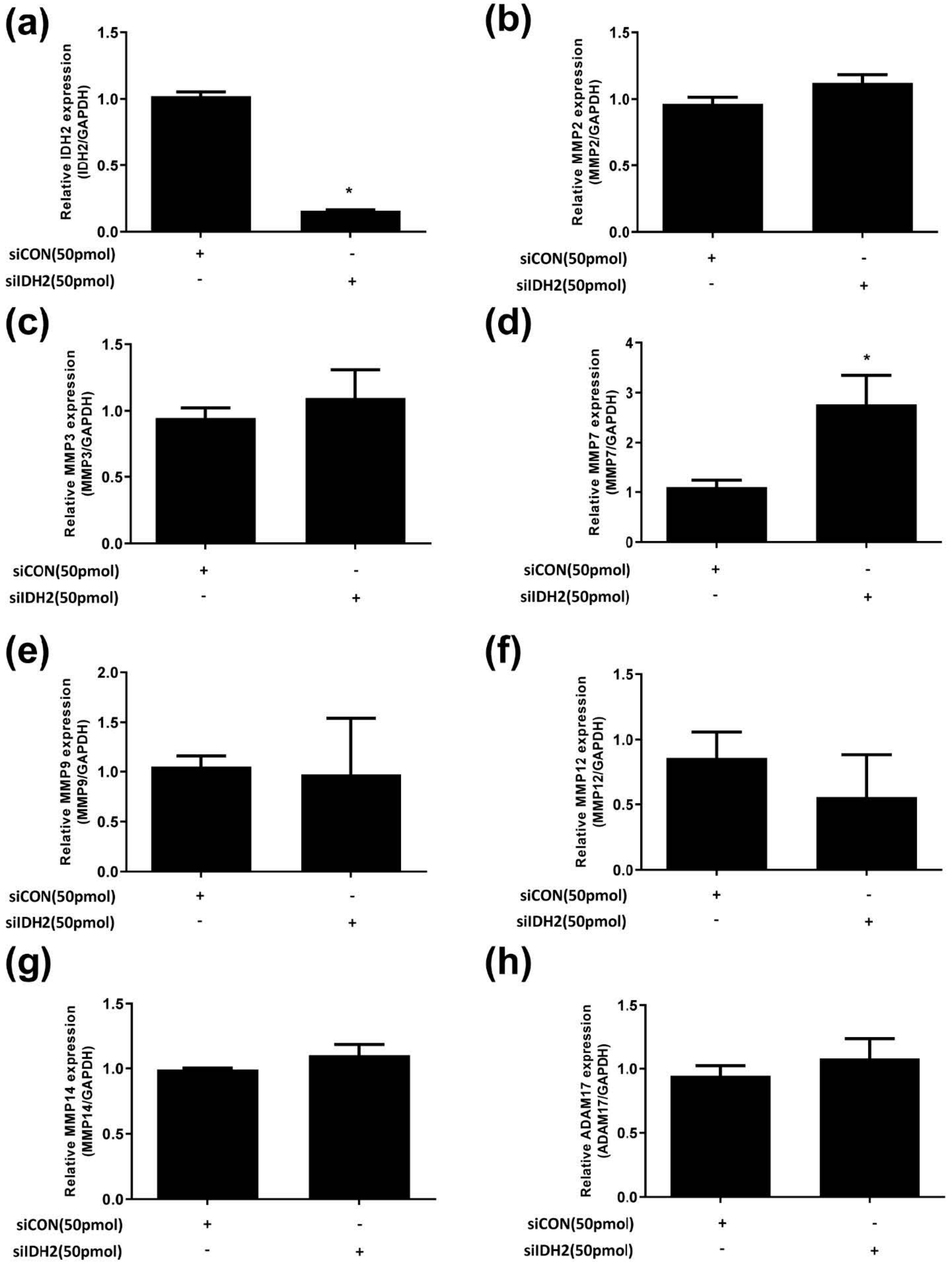

Supplementary Figure 1. Screening of MMP subtypes in HUVECs. HUVECs were transfected with 50pmol of siIDH2 and siCON for 48h. mRNA expressions of (a) IDH2 (b) MMP2 (c) MMP3 (d) MMP7 (e) MMP9 (f) MMP12 (g) MMP14 and (h) ADAM17 were quantified by qPCR. All data are presented as means ± SEM of three independent experiments, *P<0.05, compared with siCON.

Supplementary Figure S2

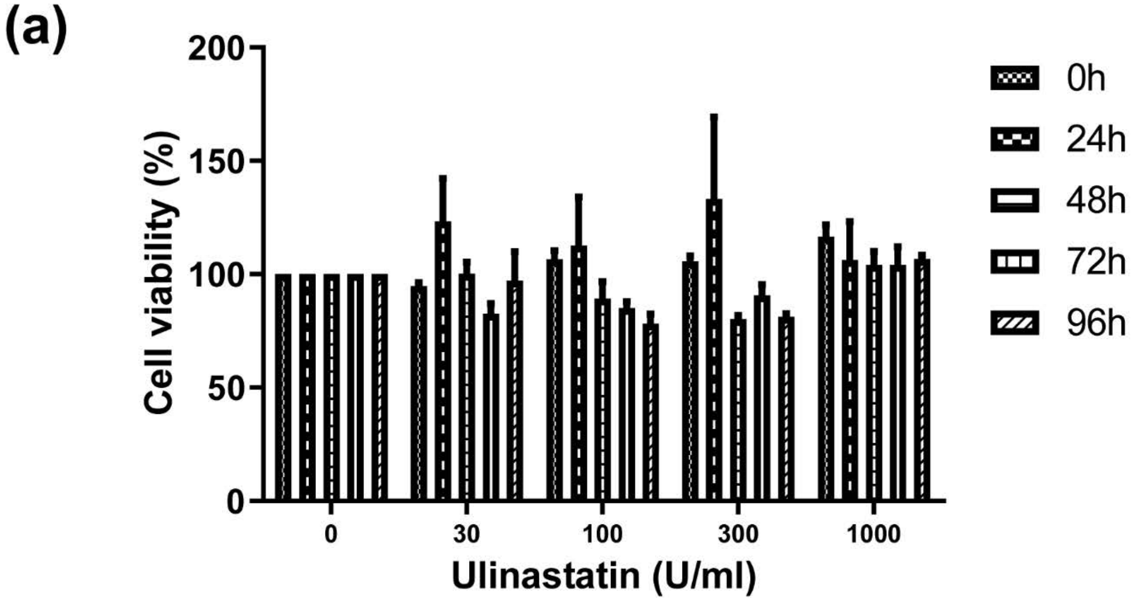

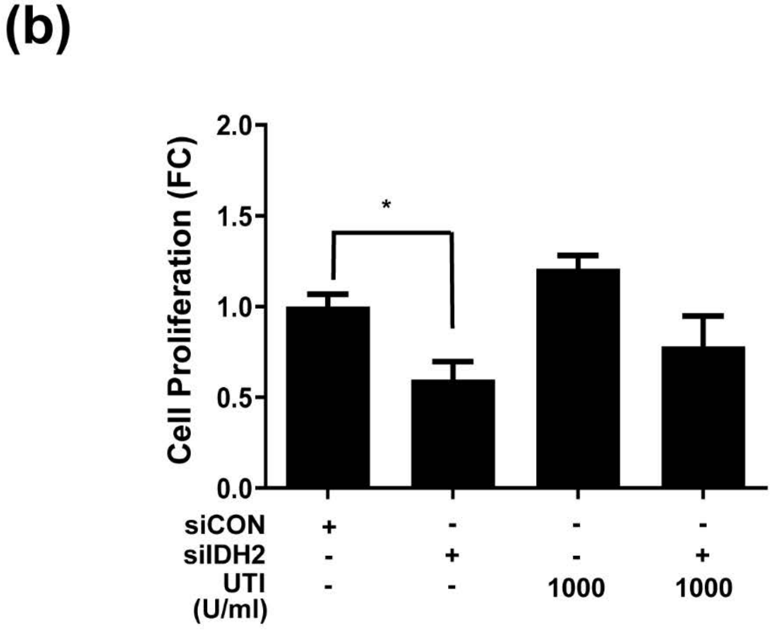

Supplementary Figure 2. Cell viability and cell proliferation after UTI treatment in HUVECs. (a) After treatment of dose dependent UTI (30, 100, 300 and 1000 U/ml) for 24, 48, 72 and 96 h in HUVECs, cell viability was measured using the ADAM-MC automatic cell counter. (b) HUVECs were transfected with 50pmol of siIDH2 and siCON with or without UTI treatment (1000 U/ml) for 48h. Cell proliferation was measured using the CCK-8 proliferation assay. All data are presented as means ± SEM of three independent experiments, *P<0.05, compared with siCON.

Supplementary Figure S3

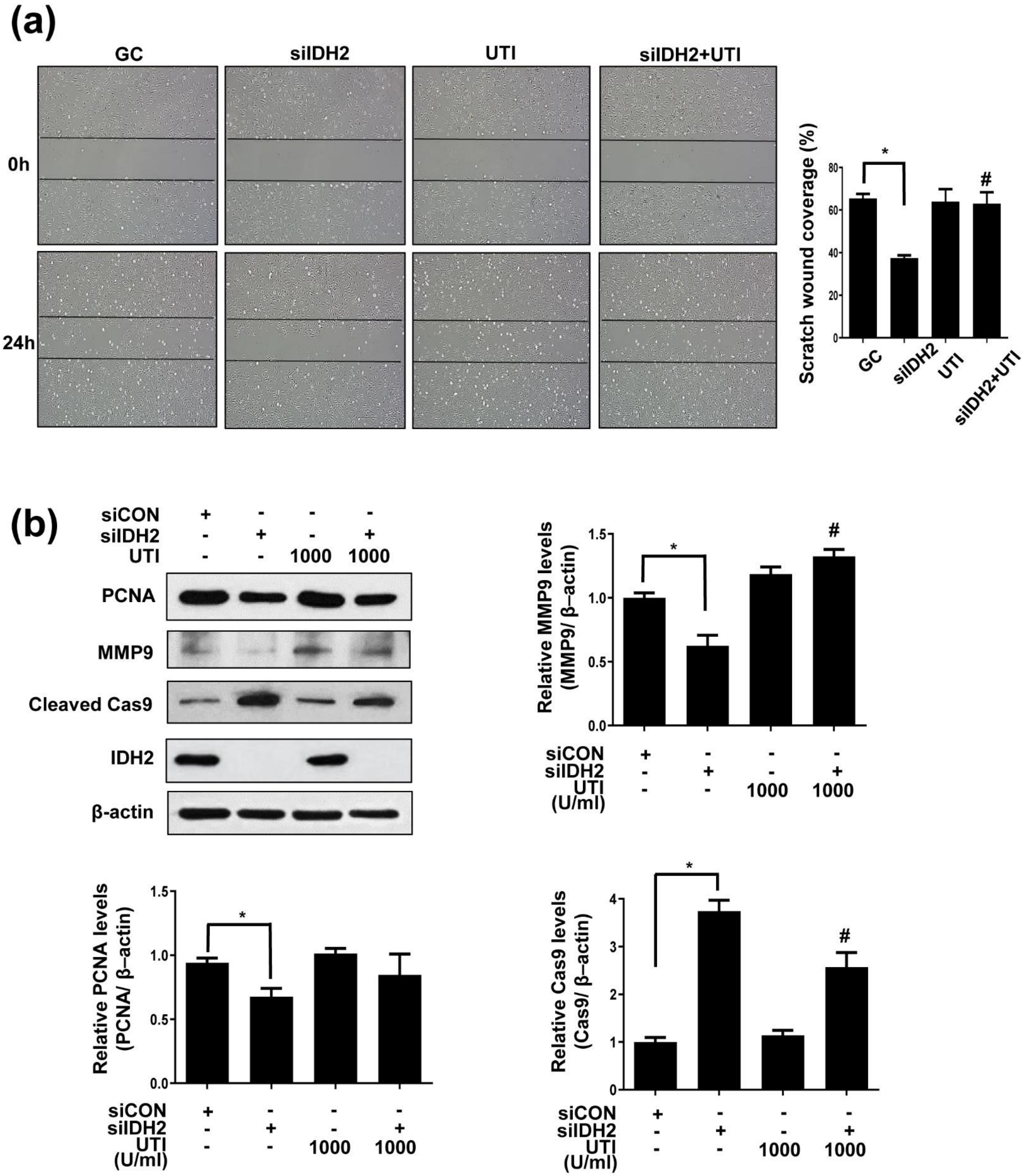

Supplementary Figure 3. Measurement of cell function after UTI treatment in HUVECs. HUVECs were transfected with 50pmol of siIDH2 and siCON with or without UTI treatment (1000 U/ml) for 48h. (a) Cell migration was assessed using the cell scratch assay. (b) Protein levels of PCNA, MMP9 and cleaved caspase 9 were detected by western blotting. β-actin was used as a loading control. Protein levels were quantified by densitometric analysis using Image-J software. All data are presented as means ±SEM of three independent experiments, *P<0.05, compared with siCON, #P<0.05 compared with the 50pmol siIDH2 treated cells.

Supplementary Figure S4

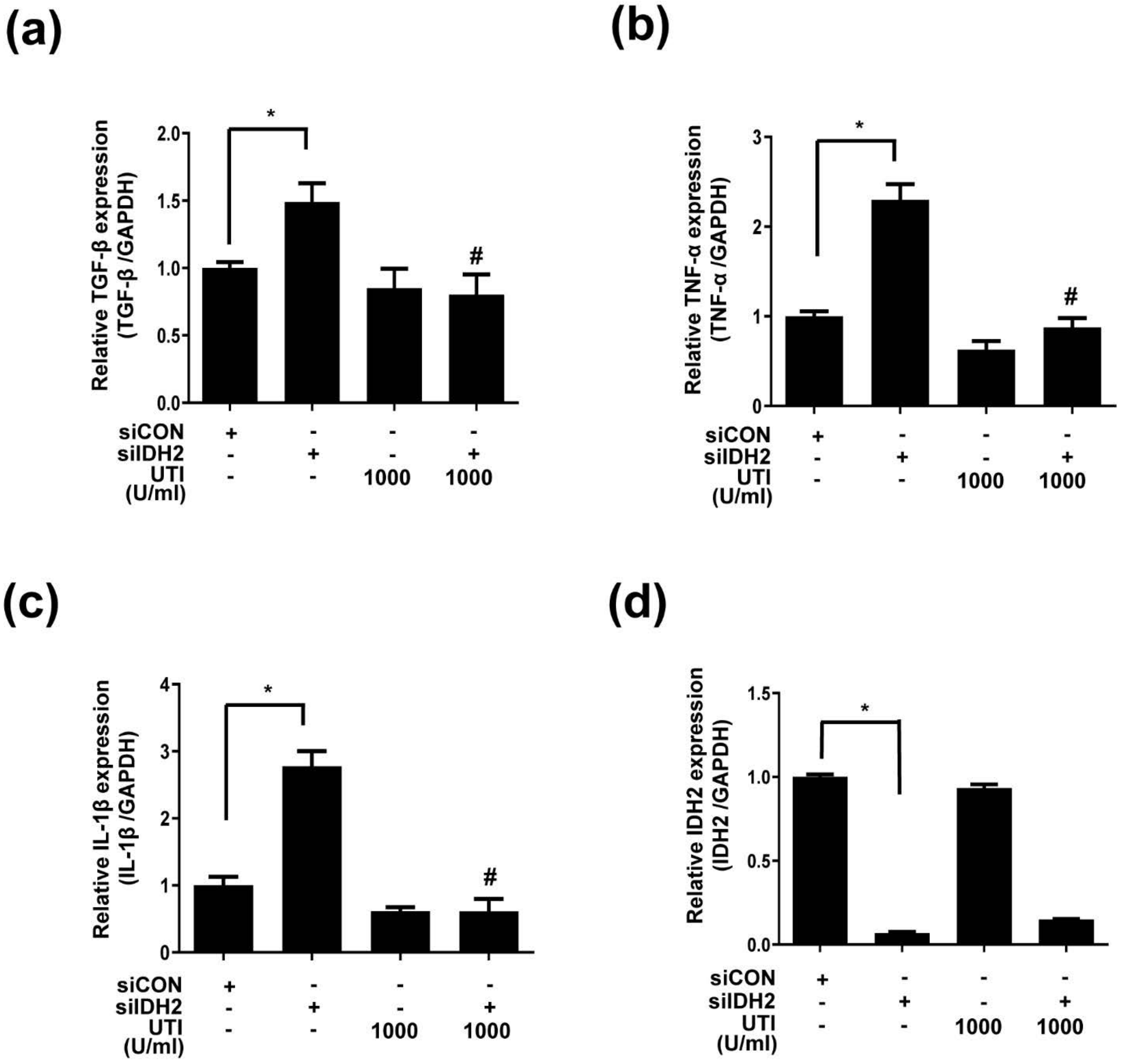

Supplementary Figure 4. Measurement of cytokines after UTI treatment in HUVECs. HUVECs were transfected with 50pmol of siIDH2 and siCON with or without UTI treatment (1000 U/ml) for 48h. mRNA expressions of (a) TGF-β (b) TNF-α (c) IL-1β and (d) IDH2 were quantified by qPCR. All data are presented as means ±SEM of three independent experiments, *$P<0.05$, compared with siCON, #$P<0.05$ compared with the 50pmol siIDH2 treated cells.
